# Noninvasive quantification of blood potassium concentration from ECG in hemodialysis patients

**DOI:** 10.1038/srep42492

**Published:** 2017-02-15

**Authors:** Cristiana Corsi, Marilisa Cortesi, Giulia Callisesi, Johan De Bie, Carlo Napolitano, Antonio Santoro, David Mortara, Stefano Severi

**Affiliations:** 1 Department of Electrical, Electronic and Information Engineering “Guglielmo Marconi”, University of Bologna, Cesena, Italy; 2 Health Sciences and Technology Interdepartmental Center for Industrial Research, University of Bologna, Cesena, Italy; 3 Mortara Instrument Inc., Milwaukee (WI), USA; 4 Molecular Cardiology, IRCCS Fondazione Salvatore Maugeri, Pavia, Italy; 5 Nephrology Dialysis, Hypertension Unit, Hospital Policlinico S.Orsola-Malpighi, Bologna, Italy

## Abstract

Blood potassium concentration ([K^+^]) influences the electrocardiogram (ECG), particularly T-wave morphology. We developed a new method to quantify [K^+^] from T-wave analysis and tested its clinical applicability on data from dialysis patients, in whom [K^+^] varies significantly during the therapy. To elucidate the mechanism linking [K^+^] and T-wave, we also analysed data from long QT syndrome type 2 (LQT2) patients, testing the hypothesis that our method would have underestimated [K^+^] in these patients. Moreover, a computational model was used to explore the physiological processes underlying our estimator at the cellular level. We analysed 12-lead ECGs from 45 haemodialysis and 12 LQT2 patients. T-wave amplitude and downslope were calculated from the first two eigenleads. The T-wave slope-to-amplitude ratio (T_S/A_) was used as starting point for an ECG-based [K^+^] estimate (K_ECG_). Leave-one-out cross-validation was performed. Agreement between K_ECG_ and reference [K^+^] from blood samples was promising (error: −0.09 ± 0.59 mM, absolute error: 0.46 ± 0.39 mM). The analysis on LQT2 patients, also supported by the outcome of computational analysis, reinforces our interpretation that, at the cellular level, delayed-rectifier potassium current is a main contributor of K_ECG_ correlation to blood [K^+^]. Following a comprehensive validation, this method could be effectively applied to monitor patients at risk for hyper/hypokalemia.

Maintenance of normal potassium homeostasis is an important clinical requirement in the treatment of several pathological conditions. As an example, in patients with acute or chronic heart failure (HF), mortality and morbidity can be reduced by the administration of drug therapies that modify potassium homeostasis. These therapies may improve clinical outcomes while, at the same time, enhancing the risk of potassium-related adverse events. The evidence is persuasive that serum potassium level should be maintained between 4.5 and 5.5 mM in patients with HF^1^,^2^. Consequently, not only accurate patient selection, but also adequate monitoring of serum potassium level, should be performed to control the benefit and risk of drug therapies in HF patients^3^,^4^.

Hypokalemia ([K^+^]<4.4 mM) was demonstrated to be an independent predictor of sudden cardiac death (SCD) in patients with HF^5^. Recent studies have shown that potassium levels outside the interval of 4.1–4.7 mM are associated with increased mortality risk^6^,^7^. The importance of potassium balance is becoming increasingly clear in patients with chronic kidney disease since, under normal circumstances, renal elimination of potassium is largely responsible for the long-term maintenance of potassium homeostasis. Patients with end-stage renal disease have a marked tendency to hyperkalemia. Haemodialysis (HD) patients have a very high incidence of cardiac events; cardiovascular diseases remain the single most common cause of death in chronic HD patients^8^,^9^. Continuous potassium monitoring could be extremely useful in order to design real-time dialysate potassium content tailored to the patient’s specific needs, or even to close the loop and personalize each single therapy session through a biofeedback system.

Electrocardiographic effects of potassium have been well known for many years^10^,^11^,^12^. Hyperkalemia first manifests in the ECG with the appearance of narrow-based, peaked T-waves, which represent the repolarization of the ventricles. In physiological conditions, they can be described by their symmetry, skewness, slope and amplitude. In pathological conditions, the shape of the T-wave may change, and measurements of those parameters could identify the onset of specific diseases. In the literature, it is reported that tall and narrow (“peaked” or “tented”) symmetrical T waves may indicate hyperkalemia^13^, whilst flat T waves may indicate hypokalemia^14^.

Following these considerations, we recently designed a method for serum potassium concentration quantification ([K^+^]) from T-wave analysis and we tested it on data from consecutive dialysis patients, since they have wide fluctuations in serum potassium pre- and post-dialysis^15^,^16^,^17^. In this study, our [K^+^] estimator was validated and tested on a large group of dialysis patients. Moreover, to give a mechanistic interpretation of the link between [K^+^] and the ECG, we hypothesized that the well-known modulation of the cardiac I_Kr_ current by extracellular [K^+^] at the cellular level could be a main contributor to the macroscopic changes of the T-wave morphology on ECG. Therefore, the estimator was tested on congenital long QT type 2 (LQT2) patients, in whom the link should be disrupted due to the presence of a genetically-determined loss of I_Kr_ current. Consequently, an artefactual hypokalemia should be apparent on the ECG. In addition, a computational model of cardiac cell electrophysiology was used to investigate the cellular basis of [K^+^]-induced T-wave modifications and support our hypothesis about the physiological processes underlying our [K^+^] estimator.

## Results

**Figure 1:**
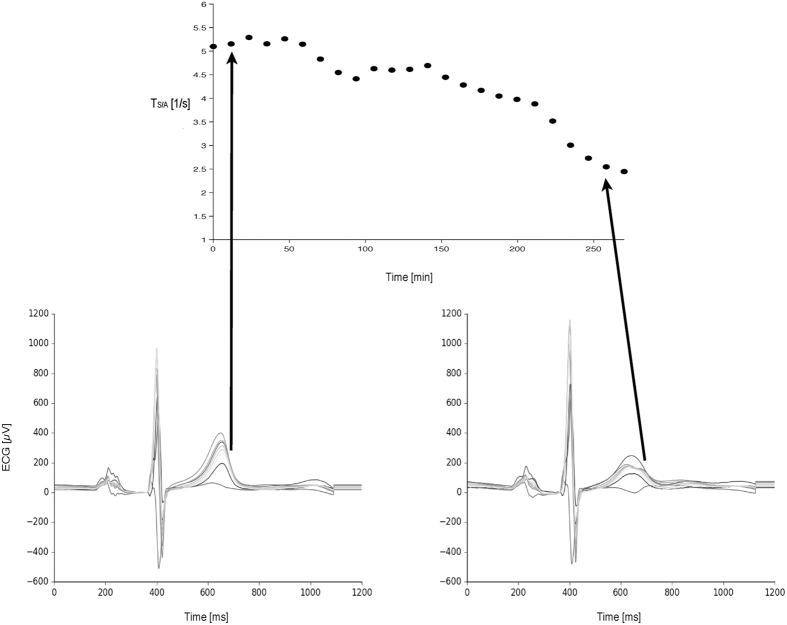
A qualitative example of ECGs (8 independent leads, from 12-lead Holter recordings) acquired in a real patient at the initial (left) and final (right) stages of a dialysis session, demonstrating the correspondence between ECG-based T_S/A_ parameter and T-wave morphology.

A qualitative example of T_S/A_ derived from different T-wave morphologies in a patient ECG is shown in Fig. 1. As expected, T-wave morphology changes significantly (Fig. 1, bottom panels) because of the large potassium removal during dialysis (Fig. 1, top panel); the slope-to-amplitude ratio reflects this modification.

Overall, we found a significant correlation (r = 0.72, p < 0.01) between T_S/A_ and [K^+^]. On the contrary, no correlation was found between T_S/A_ and heart rate, calcium, sodium, over-hydration status (actual weight-dry weight), or pH.

**Figure 2:**
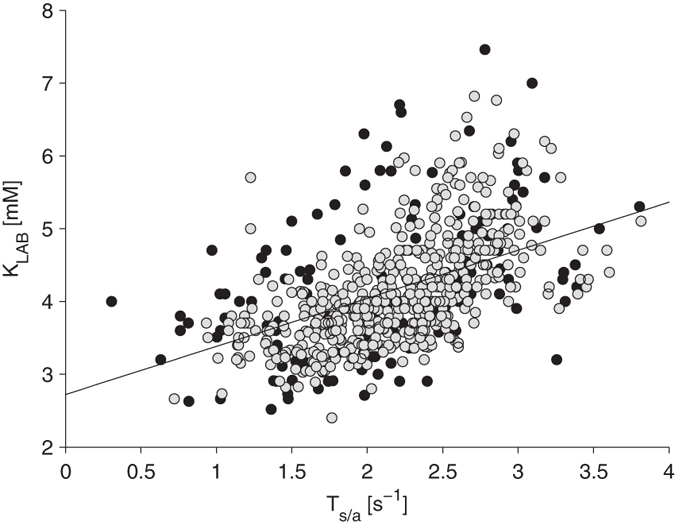
Preliminary results from the first attempt to derive an ECG-based [K^+^] estimator (K_ECG1_ = 0.66* T_S/A_+2.72) by inverting the relation between the ECG-based T_S/A_ parameter and the corresponding lab measurements of [K^+^] (K_LAB_), without any patient-specific calibration. Black dots are from sessions in which a systematic error (bias) was present all along the session, causing clustering of estimates far from the linear relationship.

**Figure 3:**
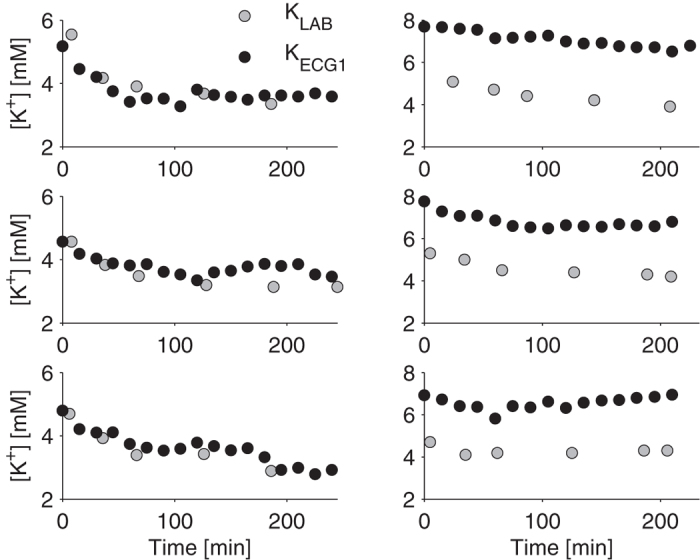
Examples from three different sessions, for two different patients: one showing good agreement (left panels) and another showing systematic error (right panels) between the K_ECG1_ (black circles) and K_LAB_ (gray circles).

Based on these results, an initial ECG-based potassium estimator was defined as K_ECG1_ = 0.66* T_S/A_ + 2.72, and compared with the reference potassium measurements (K_LAB,_ Fig. 2). Cluster analysis revealed that sessions could be divided into two groups. For one group, the agreement was quite good, with an absolute error of 0.44 ± 0.36 mM (99/128, 548 [K^+^] measurements, gray dots in Fig. 2). An example of the results obtained in one patient is shown in Fig. 3 (left panels). In the other group of 29 sessions (138 measurements, black dots in Fig. 2), the presence of a systematic error (bias) all along the session led to dramatically poorer estimates (absolute error: 0.82 ± 0.55 mM). An example of a patient with a systematic error in the [K^+^] estimates is shown in Fig. 3 (right panels).

Following a patient-specific calibration based on data from the first session (see Methods), a second estimator was identified: K_ECG2_ = 1.05*T_S/A_ + PB_2_, where PB_2_ is the patient-specific bias, with a value of 1.98 ± 0.59. The performance of the new estimator, assessed by considering all the sessions not involved in the calibration step, resulted in a mean absolute error of 0.49 ± 0.39 mM.

**Figure 4:**
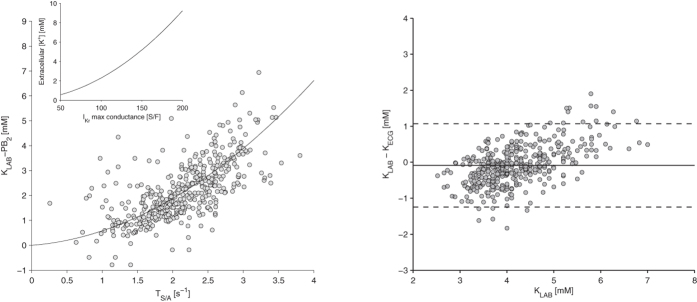
Left. When a patient-specific bias (PB_2_), estimated from the first dialysis session of each patient, is subtracted from the data, a nonlinear relationship can be observed between K_LAB_ and T_S/A_, assessed in the remaining sessions. This observation led to the definition of the final ECG-based [K^+^] estimator (K_ECG_ = 0.36*T_S/A_^2^ + 0.22*T_S/A_ + PB). This quadratic relationship is very similar to the one describing the dependence of the maximal conductance of I_Kr_ and I_K1_ currents on extracellular [K^+^] at the cellular level (inset panel^24^). **Right**: Bland-Altman plot of [K^+^] obtained applying the quadratic estimator and the reference values for [K^+^].

When the data were corrected for the patient-specific bias, a nonlinear relationship between K_LAB_ and T_S/A_ was noticed (Fig. 4, left panel); therefore, a final quadratic estimator was defined: K_ECG_ = 0.36*T_S/A_^2^ + 0.22*T_S/A_ + PB (patient-specific bias PB: 1.96 ± 0.85 mM). This equation led to a slight further improvement in the estimates (absolute error: 0.46 ± 0.42 mM).

Cross-validation using the leave-one-out approach was applied, which confirmed the previous results and led to a mean error of the estimated potassium concentration of −0.09 ± 0.59 mM and a mean absolute error of 0.46 ± 0.39 mM (Fig. 4, right panel).

For LQT2 patients, the estimator was applied using a PB equal to the PB mean value computed in the patient-specific calibration of HD patients. As hypothesized, in LQT2 patients having normal potassium levels (see K_LAB_ in Table 1) our method systematically underestimated [K^+^] (K_LAB_: 4.3 ± 0.3 mM; K_ECG_: 3.2 ± 1.1 mM, p < 0.02) except for one patient (Table 1), resulting in an error of 1.1 ± 1.3 mM. This result supports our interpretation of the mechanism linking [K^+^] and K_ECG_ (see Discussion).

**Figure 5:**
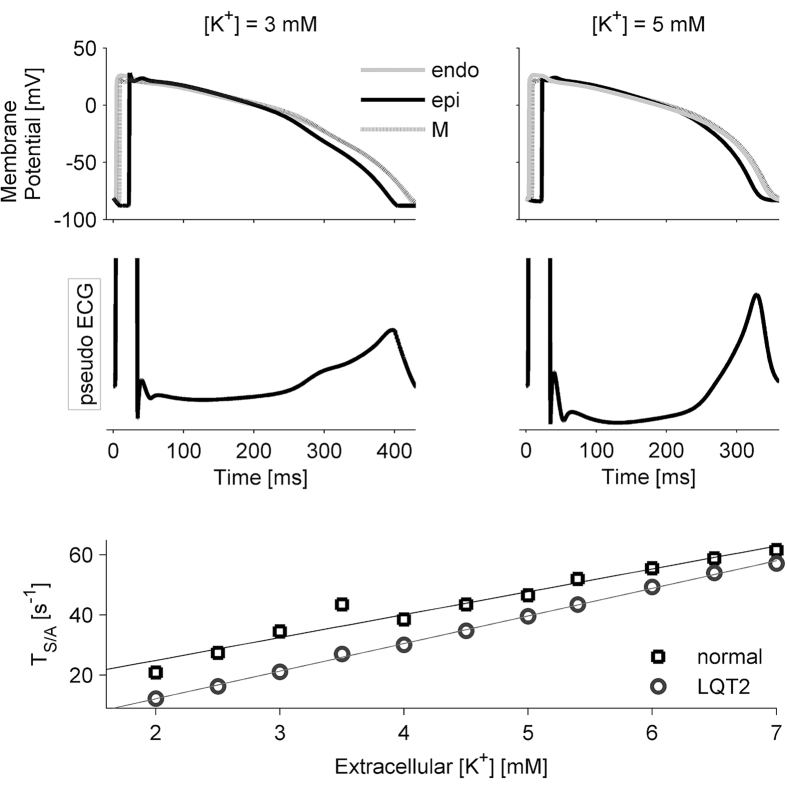
Results of the simulations performed using different potassium concentration levels. Top panels: membrane potentials for endocardial, M, and epicardial cells; middle panels: corresponding pseudo-ECGs clearly showing the differences in T-wave morphology; bottom panel: relationship between T_S/A_ and [K^+^] in control (squares) and LQT2 (circles) conditions.

The complex relation between electrical phenomena at the cellular level, involving each single cardiac cell, and their macroscopic manifestation in the ECG signal was then investigated through a computational approach (see Methods). Simulations of a cardiac tissue wedge showed that an increased extracellular potassium concentration causes action potential shortening in all cell types (endocardial (endo), M, and epicardial (epi)) but also increases the velocity of phase 3 action potential repolarization. As a consequence, the degree and timing of heterogeneity across the cardiac wall is modified (Fig. 5, top panels), and the T-wave shows an increasingly peaked shape (Fig. 5, middle panels)—quantified as an increasing value of T_S/A_ (Fig. 5, lower panel, squares) as potassium increases. Simulations of the LQT2 condition produced a generalized decrease of the T_S/A_ parameter in spite of the use of the same potassium levels used in control simulations (Fig. 5, lower panel, circles). That is, a decrease in extracellular potassium and a reduction (unrelated to potassium concentration) of I_Kr_ current have similar effects on T_S/A_.

## Discussion

The availability of a non-invasive tool for [K^+^] assessment would have a significant impact on clinical practice, facilitating cheaper and more effective monitoring of several classes of patients affected by diseases and/or undergoing pharmacological treatments that carry the risk of hypo/hyper-kalemia. We originally proposed this method for quantification of blood [K^+^] from the ECG a few years ago, with promising preliminary findings^15^. Here, we have tested the new automatic method on a total of 686 [K^+^] measurements in dialysis patients, to assess its suitability for clinical application. Moreover, we investigated the physiological mechanism underlying the link between blood [K^+^] and T-wave features in order to further evaluate the applicability of the proposed estimator.

The new method we developed was tested on data from dialysis patients. Due to the wide changes in serum potassium during the dialysis session, HD therapy is a unique “experimental model” for this testing in individual patients. Furthermore, the reference values for [K^+^], measured through standard laboratory analysis, are easily available through blood samples taken from the extracorporeal circuit. The proposed approach was tested in a population of 45 patients; we performed 686 [K^+^] measurements (pre-, post- and during HD), characterized by a wide [K^+^] range (2.5–7.5 mM). Cross-validation analysis was applied, with very stable results— indicating they are independent of the testing/validation set, at least in our population of dialysis patients.

These promising results show that K_ECG_ estimates can be an effective tool for first-level monitoring of patients at risk for hyper-/hypokalemia. The accuracy of these results makes them clinically relevant (the mean absolute error of 0.46 ± 0.39 mM can be acceptable). In fact, on the basis of the potassium estimated at the start of dialysis, the content of potassium in the dialysis bath could be adjusted in order to promote physiological reductions in serum potassium, without abrupt variations which could induce dangerous arrhythmias. Importantly, in several pathological conditions the automated estimator could be a reliable tool for [K^+^] trend monitoring, reducing the costs related to both blood sample analysis and the availability of clinical expertise to evaluate the ECG for subtle changes reflective of [K^+^] imbalance in small medical centers.

In this study a [K^+^] estimate every 15 minutes was tested, although our method does allow real-time continuous estimates by simply shifting the 2-min window in time. However, since the [K^+^] dynamic is slow, the availability of [K^+^] estimate each minute is unlikely to add clinical information.

Very recently a group from the Mayo Clinic^18^, starting from our preliminary work^15^, proposed a slightly different linear estimator based on T_slope_/√T_amp_ and tested it on patients in a dialysis setting, with similar results. Importantly, their approach did not require any patient-specific calibration. However, calibration using one blood test does not seem to be a major issue in any patient population. Indeed, this patient-specific calibration can be easily managed in HD patients, while in other patients requiring [K^+^] monitoring a blood test is always performed to better characterize each patient’s condition, and it could be used for K_ECG_ calibration. In contrast to our approach, the [K^+^] estimate was computed using the signal derived from only one precordial lead, selected from V_3_-V_6_ leads. In this study^18^ a 12-lead ECG recording is required to compute [K^+^] estimate. Some attempts (unpublished data) have been made to use a single-lead ECG, without getting comparable results. Preliminary testing using a wearable system was also unsuccessful, providing poor results (error (mM): 0.01 ± 0.82; absolute error (mM): 0.65 ± 0.49). Therefore, in our experience, the redundancy given by the 12-lead ECG does matter.

After validation, we devoted our efforts to pointing out the physiological mechanisms by which [K^+^] levels in the blood are reflected in the specific features of the T-wave on which we based our estimator. It is well accepted that T-wave amplitude and shape reflect the heterogeneous duration of the terminal repolarization phase of the cardiac ventricular action potential at the cellular level^19^, when outward potassium currents, particularly the rapid component of the delayed rectifier potassium current (I_Kr_), are the dominating currents across the cell membrane. I_Kr_ depends on potassium concentration in the extracellular space^20^,^21^—that is, in the interstitial fluid. In turn, it is usually accepted that potassium concentration in the interstitial fluid equals that in blood. Indeed, the distribution of free cations between vascular and interstitial compartments has been reported to agree with the Donnan theory^22^, which predicts a ratio between interstitial and plasma concentrations equal to 0.98^23^.

The use of a quadratic fitting relation between the actual potassium and the T_S/A_ estimator (Fig. 4, left panel) allowed us to appreciate a striking similarity between this macroscopic “black-box” relationship and a biophysically-based relationship describing molecular properties of those potassium currents that have a fundamental role in cell repolarization. Potassium ions are unequally distributed among the intracellular and extracellular spaces, being much more concentrated within the cell (about 150 mM) than in the extracellular fluid (about 5 mM). An increase of extracellular [K^+^] would be expected to decrease the potassium currents, since it leads to a reduction of the concentration gradient, hence of the electrochemical driving force for the currents themselves. On the contrary, it has been observed that some outward potassium currents, specifically I_Kr_ and I_K1_, increase in the presence of increased extracellular [K^+^] because of specific interactions between the potassium ions and the external portion of the proteins which constitute the ion channels the currents flow through. These interactions lead to a conformational change in the channel with a consequent increase in its conductance. The relation between the conductance of I_Kr_ and I_K1_ and the extracellular [K^+^] was characterized and turned out to be a quadratic function^24^. The strong similarity between the best fitting relation of our estimates and this quadratic function in its inverted form is shown in Fig. 4 (left panel inset). This merely qualitative observation suggests a key role for the modulation of potassium conductance in determining the macroscopic effects of variations in blood potassium concentration.

Based on these considerations, we tested the hypothesis that changes in I_Kr_ current due to [K^+^] changes could be a main contributor to the changes in T-wave morphology we detected with our estimator. To do so we analyzed data from LQT2 patients, in which the I_Kr_ reduction is due to a different pathological factor (a loss-of-function mutation on the gene encoding for I_Kr_ instead of hypokalemia); the estimator almost systematically produced low potassium values, as we had hypothesized. Since the analyzed patients were not hypokalemic we propose that the estimator could be a “I_Kr_-estimator”, rather than a direct potassium estimator. This possibility would explain the emerging nonlinear relationship between the indirect [K^+^] estimate and the T-wave morphology descriptor. Based on this result, a message of caution should be given: the estimation of [K^+^] from the ECG cannot be applied to patients with known alterations in I_Kr_ and/or I_K1_ or those assuming drugs affecting these cardiac ionic currents (e.g. amiodarone or sotalol).

The computational analysis provided further support for the interpretation of the mechanistic link between [K^+^] and T_S/A_. Indeed, it was possible to reproduce a positive dependence of T_S/A_ on extracellular [K^+^] when simulating a pseudo-ECG on a ventricular tissue wedge. In particular, simulations suggest the following cause-effect sequence: 1) an increase in extracellular [K^+^] leads to increases in I_Kr_ and I_K1_ (Fig. 4, left panel inset); 2) since I_Kr_ and I_K1_ are preferentially active during the late repolarization phase (phase 3), their increase leads to shortening of the action potential duration and in particular of this phase (Fig. 5, top panels); 3) the T-wave duration is shortened, but the morphology is also affected since the steeper decrease of the membrane potential causes larger potential gradients across the ventricular wall (Fig. 5, middle panel).

Notably, this dependence was qualitatively preserved when simulations were repeated with the simultaneous reduction of I_Kr_, but all the T_S/A_ estimates were shifted towards lower values. Since computational results were obtained with a specific model of human ventricular action potential^25^, in order to test their robustness with respect to this choice, we also carried out the same simulations using the O’Hara-Rudy human ventricular myocyte model^26^ and obtained similar results^27^.

The main limitation of our results concerns their applicability to patients other than those undergoing haemodialysis therapy. Indeed, specific aspects of their clinical status (e.g. possibility of previous infarction not always revealed in clinical history) or the therapeutical setting could have influenced the results, making them irreproducible in other contexts. We were careful to assess and exclude the influence of all the main possible covariates (heart rate, calcium, sodium, over-hydration status, and pH); nevertheless, the reliability of our estimator for other kinds of patients remains to be assessed.

Another potential limitation of the proposed estimator is the presence of a residual dependence of the error on the actual serum [K^+^]; larger positive errors for high [K^+^] values could lead to underestimation of severe hyperkalemic conditions. Future inclusion in the estimation process of other ECG-derived parameters known to be influenced by high [K^+^] levels (such as QRS duration or P-wave duration) could mitigate this issue.

The actual genesis of the T-wave is still a matter of debate, and a more comprehensive computational analysis of the link between [K^+^] and ECG would require modelling of the whole heart, including also (at least) apex-base and right/left ventricle heterogeneity of repolarization. Moreover, inhomogeneity of conduction due to, e.g., fibrosis should also be considered. Such complex computational modelling goes beyond the scope of the present work.

## Conclusion

The present study shows the possibility of obtaining acceptable serum potassium measurements from a digital analysis of the T wave on the ECG in HD patients. In recent years, digital technology and advanced signal processing techniques have greatly improved the quality and stability of the ECG signal, allowing researchers to further develop quantitative ECG analysis—leading to what has been referred to as an ECG renaissance.

In particular, the present results contribute to paving the way to turn a well-established clinical qualitative observation, i.e. the presence of hypo/hyperkalemic patterns in the ECG, into a new quantitative, noninvasive marker of blood potassium concentration. Whether the potassium estimation is applicable to populations other than HD patients remains to be tested, but this analysis holds promise for multiple applications and for several diseases, both in the clinical environment and in a health-monitoring scenario where it is not possible to make biochemical measurements (at home or in less well-equipped medical clinics). Importantly, a clear link with some molecular and cellular underlying processes can be established.

## Methods

### Study design

The study design included two protocols. Protocol 1 was designed to define, test and validate the ECG-based K^+^ estimator (K_ECG_) against the actual serum K^+^concentration (K_LAB_). The method we developed was tested on data from dialysis patients, since they experience large changes in blood composition, including serum potassium, during the dialysis session. In Protocol 2, in order to test the hypothesis that the main mechanism underlying the relationship between [K^+^] and T-wave is the [K^+^] modulation of the I_Kr_ potassium currents at the cellular level, we applied the estimator to patients with LQT syndrome type 2. LQT2 patients carry loss-of-function mutations of the gene encoding for HERG channels. Therefore, the I_Kr_ potassium current is reduced in a way somewhat similar to that observed in hypokalemic conditions. Based on this observation we hypothesized that if the ECG-based estimator relies on the effect of [K^+^] on I_Kr_ it should systematically underestimate blood potassium in this population.

### Data Analysis

#### Definition of the [K^+^] estimator

**Figure 6:**
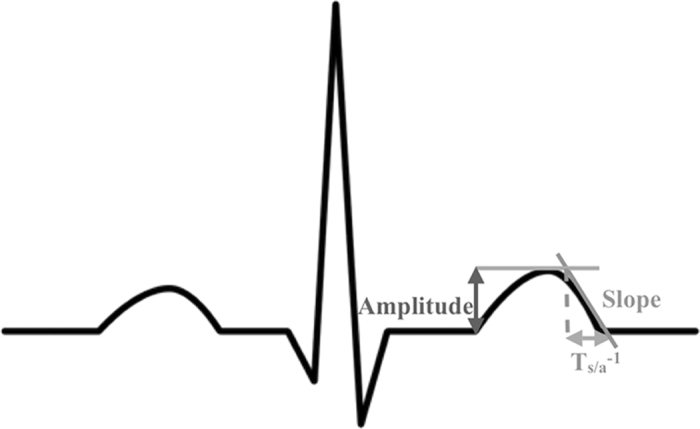
A typical QRS-T-wave complex from an idealized electrocardiogram together with the schematic explanation of the negative slope and the amplitude of the T wave used to compute T_S/A_ for [K^+^] estimation.

The earliest electrocardiographic manifestation of hyperkalemia is the appearance of narrow-based, peaked, T-waves^10^,^11^,^12^. We hypothesized that the morphological changes in the T-wave due to changes in potassium could be captured by a combination of simple measurements reflecting to what extent the T-wave is “narrow and peaked”. In other words, we were looking for an amplitude-invariant measurement of the sharpness of the T-wave. We focused our analysis on the ratio of the T-wave downslope-to-amplitude (T_S/A_, Fig. 6). Based on the relationship between T_S/A_ and serum [K^+^], we defined an estimator from which the [K^+^] values can be derived.

We retrospectively analyzed 12-lead Holter ECG recordings (H12 + , Mortara Instrument Inc.). The most significant two eigenleads, associated with the first two eigenvalues, were used to calculate the downslope and the amplitude of the T-wave for each beat. The ECG-based potassium estimator (K_ECG_) was defined as a quadratic function of the median value of T_S/A_, automatically computed over a 2-minute window at intervals of 15 minutes.

ECG data were exported and analyzed by implementing a dynamic-link library that interfaces to the post-processing software already available from Mortara Instrument Inc. (SuperECG/Spectrum Mortara Instrument Inc.).

#### Protocol 1: Validation of the [K^+^] estimator

We analyzed ECG recordings acquired during 128 dialysis sessions in 45 HD patients (once-a-week sessions for three weeks, the same day of the week, each session about four hours long). During each dialysis session, for the majority of sessions, six gold standard [K^+^] measurements (K_LAB_) were obtained (for a total of 686 measurements) from blood samples (RapidLab 855, Bayer and GemPremier, Instrumentation Laboratory) at the following times: 0, 30, 60, 120, 180, 240 minutes from the start of dialysis. Na^+^, Ca^2+^, and Mg serum concentrations, weight loss, and heart rate were assessed as well.

Patients were enrolled from Malpighi Hospital in Bologna (41 patients) and Infermi Hospital in Rimini (4 patients) within a previous study on haemodialysis therapy^28^; approval of the Institutional Review Board (IRB) Ethics Committee was not required. All the procedures were in accordance with the Helsinki Declaration. The main characteristics of the study population are shown in Table 2. Patients were eligible if they had been at a metabolic steady state in dialysis treatment for at least 6 months with thrice-weekly and double-needle haemodialysis. Exclusion criteria were: anaemia, history of myocardial ischemia, coronary bypass, atrial fibrillation.

In this patient population the relation between T_S/A_ and K_LAB_ was modeled with a quadratic function. In order to compensate for eventual systematic error, the first session of each patient was used to calibrate the estimate and thus was excluded from the subsequent analysis. The corrective term was computed as the mean of the differences between the T_S/A_ estimates and K_LAB_ values in the first and last measurements of the first session (see Results). The estimator was then validated on the data of the second and third sessions of each patient.

#### Protocol 2: Serum [K^+^] and T-wave relationship

We analyzed the [K^+^] estimator on 12 patients (5 men; age: 25 ± 15 years) with LQT syndrome type 2. In each patient, [K^+^] measurement (K_LAB_) was obtained immediately before resting ECG recordings (12-lead Holter H12 + , Mortara Instrument Inc.) from blood samples (RapidLab 855, Bayer). Patients were enrolled from Salvatore Maugeri Foundation in Pavia.

### Statistical Analysis

In Protocol 1, regression analysis was applied to assess the correlation between the T-wave descriptor and serum [K^+^] (and other dialysis-related factors). Sessions under study were then clustered into two groups based on the amount of systematic estimation bias, using the k-means algorithm^29^. This is an iterative procedure that partitions the provided data into a given number of clusters—two in this case, minimizing the distance between each point and the centroid of the cluster.

We applied the Leave One Out cross validation by excluding data obtained from one patient from the validation-set at each iteration. The Bland-Altman analysis was performed and the absolute error was also computed between K_ECG_ and K_LAB_ for each patient.

In Protocol 2, a paired t-test was applied to assess the presence of a significant bias between K_ECG_ and K_LAB_ in LQT2 patients. All statistical analysis was performed using Matlab 11a (MathWorks, Inc.).

### Computational Modelling

The relationship between [K^+^] and T-wave was analyzed in silico using the well-established approach^30^,^31^,^32^,^33^,^34^ of simulating the electrical propagation in one-dimensional virtual cardiac tissue. The action potential (AP) propagation was reconstructed in a theoretical fibre 1.65 cm long, composed of 165 cells connected through gap junctions. This reconstruction represents the broad planar wavefront that propagates from endocardium to epicardium during normal ventricular excitation^35^ as a result of Purkinje network participation. The model also represents AP propagation in the arterially perfused transmural wedge preparation, used in particular by Antzelevitch and colleagues in experimental *in vitro* studies of ECG waveforms^19^,^36^,^37^.

The electrical activity of each single myocardial cell in the fibre was simulated by means of a human ventricular myocyte computational model^25^, in which the action potential is numerically constructed on the basis of experimental data. Included in the model are all the main membrane ionic-channel currents, mathematically represented by a Hodgkin-Huxley type formalism, as well as ionic pumps and exchangers participating in the AP. In addition, processes that regulate ionic concentration changes, especially dynamic changes of intracellular calcium concentration, were introduced. Electrical heterogeneity simulating endo and epi APs was simulated according to the original publication, by changing a few parameters in the formulation of the transient outward K current, I_to_. Ionic current modifications were incorporated into the model, based on available experimental data, to reproduce human M-cell action potentials: I_Ks_ and I_K1_ current conductances were reduced by 54% and 26%^38^,^39^, respectively, and a late sodium current was added by introducing a non-inactivating percentage (0.9%) of the fast I_Na_ current^40^. Midmyocardial (mid) cells were modelled with intermediate parameter values (between those of endo and epi). Based on the experimental characterization of the transmural heterogeneity by Glukhov *et al*.^41^ through optical mapping of the coronary-perfused left ventricular wedge preparations from human nonfailing hearts, the theoretical fibre was composed of 25 endo, 25 M, 50 mid, and 65 epi cardiac cells, serially arranged.

Intracellular and extracellular conductivities were homogeneous throughout the fibre and set to 0.65 and 2.55 mS/cm, respectively, resulting in a conduction velocity of 44 cm/s recorded both *in vivo* and across an arterially perfused transmural wedge preparation (average thickness of 1.29 ± 0.15 cm)^37^. A suprathreshold current stimulus was applied to cell #1 to initiate AP propagation from endocardium to epicardium. The pseudo-ECG signal was computed as previously described^30^,^31^:


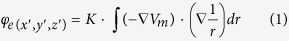


where *∇V*_*m*_ is the spatial gradient of the transmembrane potential, *K* is a constant including the radius of the fibre and the ratio between intracellular and extracellular conductivity, and *r* is the distance from a source point (x,y,z) to a field point (x′,y′,z′). The variable *φ*_e_, computed at an “electrode” site 2.0 cm away from the epicardium along the fibre axis, constitutes the ECG waveforms we reported.

The effects of altered extracellular [K^+^], from 2–7 mM, were analysed. The LQT2 condition was simulated by blocking 50% of the I_Kr_ current. Model equations were implemented in Matlab 11a (The MathWorks Inc.) and translated into cellML language using the COR environment. Mono-domain equations were solved using Chaste Software^42^.

## Additional Information

**How to cite this article**: Corsi, C. *et al*. Noninvasive quantification of blood potassium concentration from ECG in hemodialysis patients. *Sci. Rep.* **7**, 42492; doi: 10.1038/srep42492 (2017).

**Publisher's note:** Springer Nature remains neutral with regard to jurisdictional claims in published maps and institutional affiliations.

## Figures and Tables

**Table 1:** Results obtained from LQT2 patients (K_LAB_: serum potassium concentration from blood test; K_ECG_: ECG-based serum potassium concentration estimate; m: mean; sd: standard deviation).

Patient #	Age	Gender	K_LAB_ [mM]	K_ECG_ [mM]
1	26	F	4.6	2.2
2	57	F	3.9	2.5
3	39	F	4.5	2.5
4	11	F	4.8	3.1
5	47	F	4.4	4.2
6	36	F	4.2	2.4
7	39	M	4.7	2.8
8	26	M	4.0	2.9
9	19	M	4.1	2.8
10	17	F	3.9	6.3
11	17	M	4.2	4.0
12	12	M	3.9	2.7
m ± sd			4.3 ± 0.3	3.2 ± 1.1

**Table 2:** Patient characteristics.

Age (*years*)	65 + 13
Male/Female	23/22
Dialytic Age (*months*)	60 + 48
Dry weight (*kg*)	68.6 + 14.3
BMI (*Kg/m*^*2*^)	26.1 + 4.7
Primary renal pathology	Vascular disease from renal hypertension (10), Vascular disease from renal malignant hypertension (9), Nephroangiosclerosis (3), Adult type (dominant) polycystic kidney disease (3), Nephropathy from lga, demonstrated by immunofluorescence (1), Glomerulonephritis (9), Renal tumor (2), Glomerulosclerosis (5), Chronic pyelonephritis (3)
Co-morbidity	Diabetes (10), Hypertension (18), Stenosis or aortic insufficiency (7), Obesity (1), Hypertensive Cardiomyopathy (1), Colon diverticulosis (2)
